# Molecular Mechanisms of Metabolic Syndrome

**DOI:** 10.3390/ijms25105452

**Published:** 2024-05-17

**Authors:** Cosmin Mihai Vesa, Dana Carmen Zaha, Simona Gabriela Bungău

**Affiliations:** 1 Doctoral School of Biomedical Sciences, University of Oradea, 410087 Oradea, Romania; cosmin.vesa@csud.uoradea.ro (C.M.V.); danaczaha@gmail.com (D.C.Z.); 2 Department of Preclinical Disciplines, Faculty of Medicine and Pharmacy, University of Oradea, 410073 Oradea, Romania; 3 Department of Pharmacy, Faculty of Medicine and Pharmacy, University of Oradea, 410087 Oradea, Romania

Metabolic syndrome represents a cluster of conditions, such as abdominal obesity, hypertension, dyslipidemia, and hyperglycemia, that are highly prevalent in developed countries because of unhealthy lifestyles [1]. Metabolic syndrome presence greatly increases the risk of cardiovascular diseases, such as myocardial infarction or stroke, contributing to the morbidity and mortality of affected individuals [2,3]. Complex pathogenetic pathways, such as insulin resistance, endothelial dysfunction, oxidative stress, inflammation, and altered lipid metabolism, are involved in metabolic syndrome [4]. Although numerous evaluations have been performed to explore these mechanisms, complex intracellular pathways still need to be investigated [5]. A better understanding of the role of adipokines [6], GLP-1 peptide [7], miRNAs [8], and pro-inflammatory interleukins [9] can provide a better insight into this pathology. These discoveries will be useful in developing novel drugs addressing metabolic syndrome and its components [10].

The summary below presents the most important results obtained by researchers who published articles in our Special Issue.

Sodum N. et al. demonstrated that combinations of certain amino acids can increase GLP-1 secretion in mouse enteroendocrine STC-1 cells (Contribution 1). Bungau A.F. et al., by analyzing 389 patients diagnosed with acne vulgaris, demonstrated that metabolic preconditioning, autoimmune thyroiditis, and hypothyroidism are significant risk factors for the severity of acne. Moreover, the association between metabolic preconditioning (MPG) and endocrine preconditioning (EP) led to more severe acne compared to MPG or EP alone, demonstrating complex physio-pathological pathways interconnecting less-explored pathologies, such as dermatological ones (Contribution 2). Another important contribution was that of Tonyan Z.N., who demonstrated that an increased level of miR-5588-5p, miR-125b-2-3p, and miR-1284 as well as a reduced level of miR-496 represent the presence of type 2 diabetes and are not seen in individuals without diabetes, opening the way for developing the genetic signature of metabolic syndrome components (Contribution 3).

Another biochemical characteristic of metabolic syndrome was discovered by Rigamonti A.E., who demonstrated that a cluster of 15 sphingolipids can differentiate between individuals at a normal weight, individuals with obesity and metabolic syndrome, and individuals with obesity and without metabolic syndrome (Contribution 4). 

Todosenko N. et al. reviewed the role of mitochondrial AAA + protease Lon (Lonp1) as a potential target for metabolic syndrome because mitochondrial dysfunction is a pathogenetic component of metabolic syndrome (Contribution 5).

Research needs to be continued in this thematic area because, as seen above, cellular and genetic modifications in metabolic syndrome can serve as diagnostic or therapeutic modalities for improving the outcomes of patients with this syndrome.
